# Comparison of the Number of Image Acquisitions and Procedural Time Required for Transarterial Chemoembolization of Hepatocellular Carcinoma with and without Tumor-Feeder Detection Software

**DOI:** 10.1155/2013/580839

**Published:** 2013-07-29

**Authors:** Jin Iwazawa, Shoichi Ohue, Naoko Hashimoto, Takashi Mitani

**Affiliations:** ^1^Department of Radiology, Nissay Hospital, 6-3-8 Itachibori, Nishi-ku, Osaka 550-0012, Japan; ^2^Department of Radiology, Komatsu Hospital, 11-6 Kawakatsucho, Neyagawa 572-8567, Japan

## Abstract

*Purpose*. To compare the number of image acquisitions and procedural time required for transarterial chemoembolization (TACE) with and without tumor-feeder detection software in cases of hepatocellular carcinoma (HCC). *Materials and Methods*. We retrospectively reviewed 50 cases involving software-assisted TACE (September 2011–February 2013) and 84 cases involving TACE without software assistance (January 2010–August 2011). We compared the number of image acquisitions, the overall procedural time, and the therapeutic efficacy in both groups. *Results*. Angiography acquisition per session reduced from 6.6 times to 4.6 times with software assistance (*P* < 0.001). Total image acquisition significantly decreased from 10.4 times to 8.7 times with software usage (*P* = 0.004). The mean procedural time required for a single session with software-assisted TACE (103 min) was significantly lower than that for a session without software (116 min, *P* = 0.021). For TACE with and without software usage, the complete (68% versus 63%, resp.) and objective (78% versus 80%, resp.) response rates did not differ significantly. *Conclusion*. In comparison with software-unassisted TACE, automated feeder-vessel detection software-assisted TACE for HCC involved fewer image acquisitions and could be completed faster while maintaining a comparable treatment response.

## 1. Introduction

Two randomized trials have shown that transarterial chemoembolization (TACE) confers significant survival benefits [1, 2]. It has subsequently been accepted as a standard locoregional therapy for managing unresectable hepatocellular carcinoma (HCC). Detection of tumor feeders using intraprocedural imaging is indispensable for the technical success of this procedure. However, sequential angiographic acquisitions are usually necessary to accurately determine the feeder vessels in manual assessments using two-dimensional (2D) angiography. Additional angiographic runs at different angles are often required in patients with highly complex hepatic arterial vasculature. Such efforts are time-consuming and increase radiation exposure and contrast material use.

A software program specifically designed to assist in planning selective liver tumor embolization (FlightPlan for Liver, GE Healthcare, Waukesha, WI, USA) was recently developed to detect and visualize potential tumor feeders from three-dimensional (3D) C-arm computed tomography (CT) data [3]. When catheter entry and a target tumor are chosen on the multiplanar reformatted (MPR) C-arm CT images, the software automatically predicts tumor feeders by showing a color-coded image on the workstation screen. With only a single acquisition of a nonselective C-arm CT during contrast injection from the proximal hepatic artery, the software then determines each intrahepatic tumor feeder supplying every liver tumor. Therefore, software-assisted TACE is theoretically expected to reduce the number of total image acquisitions and overall procedural time by sparing unnecessary catheterizations. 

In this study, we compared the number of image acquisitions and overall procedural times required for TACE with and without assistance of the automated feeder-vessel detection software in cases of HCC.

## 2. Materials and Methods

### 2.1. Patients

We retrospectively reviewed the records of patients with HCC who came to Nissay Hospital and underwent lipiodol-based TACE using a C-arm cone-beam angiographic system between January 2010 and February 2013. Patients with diffuse and infiltrative HCC or an extrahepatic supply to the tumor were excluded from this study. The study group comprised 134 patients. Fifty patients (September 2011–February 2013) were treated with the assistance of the tumor-feeder detection software, whereas 84 patients (January 2010–August 2011) received treatment without software assistance. 

This study was conducted in accordance with the guidelines of our Institutional Review Board. Before TACE, each patient provided written, informed consent for the use of the software.

### 2.2. TACE without Software Assistance

Angiographic procedures were performed by one of two board-certified interventional radiologists who used a flat panel detector C-arm angiographic system (Innova 3100, GE Healthcare). First, a 4-Fr catheter was placed in the celiac artery via the femoral artery. A right or left hepatic artery angiogram was subsequently obtained through a coaxially inserted microcatheter. If the hepatic arteries overlapped on the frontal angiogram, an additional angiogram in a different angle was further obtained. If the tumor stain was obscure on the angiogram, an additional C-arm CT image was acquired during contrast injection from the same hepatic artery. 

After the radiologists confirmed the target tumor, 1.7- to 2.7-Fr microcatheters were advanced into the suspected tumor feeders. Angiograms and C-arm CT images were obtained from the suspected feeder artery to confirm whether the target tumor was located within the treatment area. If the investigated feeder artery was not the true feeder vessel, the microcatheter was replaced into the second most probable arterial branch. When multiple tumors existed, the same procedure for each target tumor was attempted. After confirming the true feeder artery, the hepatic areas containing the target tumors were infused with an appropriate dose of chemotherapeutic agents mixed with lipiodol (Lipiodol Ultrafluid; Terumo, Tokyo, Japan) and embolized with gelatin particles (Gelpart; Nippon Kayaku, Tokyo, Japan) until the tumor vessels were completely filled. Postprocedural C-arm CT images were obtained to ensure that no viable tumors or additional tumor feeders remained. 

### 2.3. TACE with Software Assistance

After performing celiac angiography using a 4-Fr catheter, a microcatheter (2.0–2.7 Fr) was inserted coaxially into the common or proper hepatic artery. Nonselective C-arm CT images from the artery were subsequently obtained. C-arm CT data sets were automatically transferred to the external workstation (Advantage Workstation 5.0; GE Healthcare) in which a 3D hepatic arterial tree was constructed for further vascular mapping. By using the imaging data of nonselective C-arm CT, software analysis was attempted to determine tumor feeders for all targets. In patients with many tumor burdens (generally 5 or more lesions in the treatment area), the tumor feeder was extracted using the software only for the largest tumor. 

After the software indicated the tumor feeder, 1.7-Fr to 2.7-Fr microcatheters were advanced into the suggested tumor feeders, regardless of whether the vessel was a feeder. A C-arm CT image of the probable tumor feeder was acquired during the contrast injection to confirm whether the vessel supplied the target tumor. The radiologists confirmed the feeder vessel when the target tumor was enhanced on the C-arm CT by referencing the pretreatment CT or magnetic resonance images. The hepatic areas containing target tumors were subsequently chemoembolized, as described previously. If the suggested vessels did not supply the target, the second most probable feeder was determined manually by using the previously obtained angiograms and C-arm CT images. Postprocedural C-arm CT images were then obtained to ensure that no viable tumors or additional tumor feeders were missed. 

### 2.4. C-Arm Computed Tomography

C-arm CT image was acquired with the following parameters: total scanning angle, 200°; rotation speed, 20°/s or 40°/s; acquisition time, 5 s; matrix size, 1500 × 1500; isotropic voxel size, 0.2 mm; and effective field of view, 18 cm^2^. Vascular images for software analysis were obtained by injecting 10–15 mL of iopamidol at a flow rate of 1.0–1.5 mL/s into the common or proper hepatic artery, depending on the perfusion area. Contrast material flowed for 10 s; therefore, the total volume injected depended on the flow rate. Data acquisition started 7-8 s after the intra-arterial injection of contrast material. Volume data sets were transferred to an external workstation (Advantage Workstation 4.2 or 5.0; GE Healthcare) in which the images were reconstructed in multiple planes. The size and number of tumors were determined from the C-arm CT images acquired during each TACE session.

### 2.5. Software Analysis

Detection of tumor feeders utilizing the software was performed during the angiographic session by a single radiology technologist who had more than 30 years' experience in angiographic image acquisition. All image analyses related to tumor-feeder detection were performed on the same commercial workstation (Advantage Workstation 5.0; GE Healthcare). After acquiring the C-arm CT data, the technologist chose the catheter entry site on the MPR images. By using the software's extraction function, the arterial vasculature and all the tumors were displayed as a circumscribed image on the 2D MPR in approximately 15 s. The technologist subsequently placed a circular region-of-interest (ROI) on the target tumor in the 2D MPR images to cover the entire tumor. Then, with the extraction function, the software analyzed the most probable tumor feeders that connected the selected catheter entry to the target region and displayed color-coded images in approximately 30 s. The total processing time required—from the time the C-arm CT image was available to the time when the technologist completed the tumor-feeder detection with the software—was approximately 2 min.

### 2.6. Treatment Evaluation

The therapeutic efficacy of TACE was evaluated on the basis of the change in the maximum diameter of the viable portion of the target lesions, as observed on triphasic contrast-enhanced CT images acquired 1–3 months after therapy. Recurrence was confirmed by evidence of an abnormal early enhancement in arterial phase images from contrast-enhanced CT. The response was evaluated according to the modified Response Evaluation Criteria in Solid Tumors [4]. The response categories were complete response (CR; disappearance of any intratumoral arterial enhancement), partial response (PR; at least a 30% decrease in the sum of the diameters of viable lesions), stable disease (SD; any cases that did not qualify for the other 3 categories), and progressive disease (PD; at least a 20% increase in the sum of the diameters of viable lesions). Objective response (OR) was defined as the sum of the cases that were categorized as CR and PR. The areas of lesions in which lipiodol uptake was observed were considered as necrotic tissue.

### 2.7. Data and Statistical Analyses

One interventional radiologist who participated in all angiographic procedures performed the data analysis. The number of image acquisitions and the overall procedural time were assessed, based on the patient record chart and on data from the picture archiving and communication system (PACS). The images included the first celiac artery angiogram to the last posttreatment C-arm CT. The overall procedural time of a single TACE session was the time between the insertion and the removal of a 4-Fr sheath. The Mann-Whitney *U* test or Fisher's exact test was used to compare the parameters of both study groups. All tests were two-sided. Factors were considered statistically significant with a *P* < 0.05.

## 3. Results 

### 3.1. TACE

**Figure 1:**
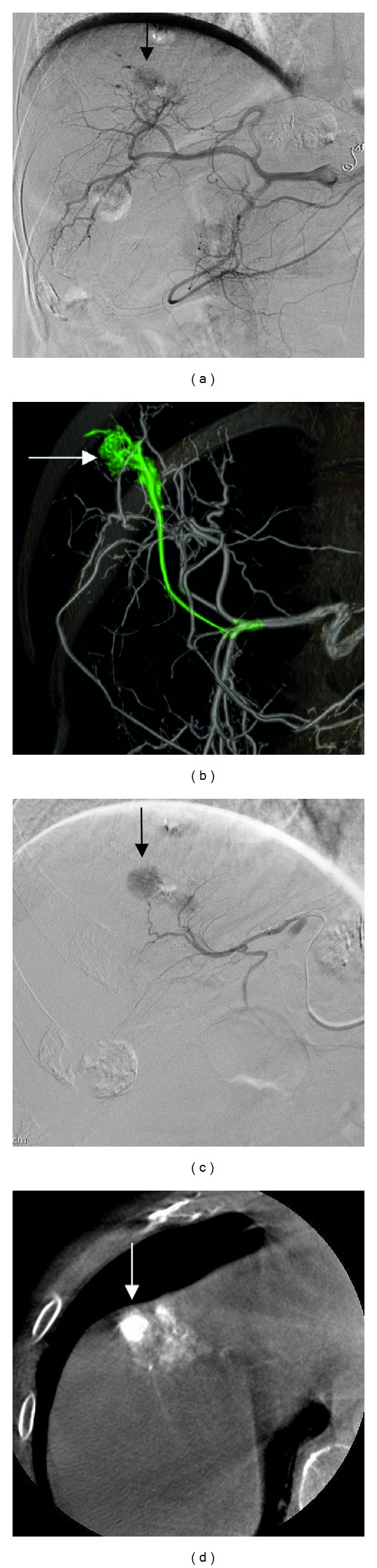
Images from a patient with hepatocellular carcinoma undergoing transarterial chemoembolization with the assistance of automated feeder-vessel detection software. (a) The common hepatic artery angiogram shows the target tumor (arrow) at the right hepatic dome. Identifying the complex arterial vasculature of the tumor feeder is difficult. (b) The volume-rendered C-arm computed tomography (CT) image, showing the extracted tumor feeder by the software as a path from the catheter to the target (arrow) indicated in green. (c) Selective catheterization directly into the suggested feeder artery, based on the software analysis, shows tumor enhancement (arrow). (d) The axial C-arm CT image, obtained during contrast injection from the same feeder artery, confirms target enhancement (arrow) in association with the treatment area.

Table 1 summarizes the baseline characteristics of the patients who underwent TACE with and without software assistance. There were no significant differences between the study groups in any of the factors investigated. We initially performed TACE for 84 patients in 134 hepatic areas without software assistance and for 50 patients in 87 hepatic areas using the software.  Figure 1 shows a representative case treated with software-assisted TACE.

### 3.2. The Number of Image Acquisitions

Table 2 lists the mean number of angiographies and C-arm CT image acquisitions in a single session of TACE. The number of angiography acquisitions per session reduced significantly with the assistance of software, irrespective of the patient's tumor multiplicity. The overall angiography acquisition reduced from 6.6 times to 4.6 times with the assistance of software (*P* < 0.001). The number of C-arm CT acquisitions per session did not differ between the groups. Total image acquisition significantly decreased from 9.7 times to 7.1 times with the use of software analysis in TACE for a single lesion (*P* = 0.001). For multiple lesions, total image acquisition reduced from 11.0 times to 9.6 times with the software; however, the difference did not reach a statistical significance. Overall image acquisitions per session significantly reduced from 10.4 times to 8.7 times with the software (*P* = 0.004). 

### 3.3. Procedural Time

Table 3 shows the mean procedural time needed for single-session TACE with and without software assistance. The overall procedural time significantly lowers with software assistance than without it (103 min versus 116 min, *P* = 0.021). The procedural time was significantly lower in TACE for a single lesion (88 min versus 109 min, *P* = 0.013), but the difference was not significant for multiple lesions.

### 3.4. Treatment Response

The therapeutic efficacies of the study groups are listed in Table 4. There were no significant differences in the overall treatment response between the 2 groups (*P* = 0.728). A CR was obtained in 34 (68%) patients and 53 (63%) patients who received TACE with and without software assistance, respectively. An OR was obtained in 39 (78%) patients and 68 (80%) patients who received TACE with and without software assistance, respectively. No significant differences were found in the CR (*P* = 0.565) or OR (*P* = 0.680) rates between the 2 groups.

## 4. Discussion

The reported sensitivity in detecting tumor feeders using the software varies from 80% to 93% [3, 5–7]. All previous studies suggested that the software more accurately enables the identification of tumor feeders than that achieved with a manual assessment using angiography. Furthermore, the processing time required for detecting tumor feeders with the software ranged from 135 s to 142 s [3, 6]. The time necessary for a software analysis was significantly shorter than the time for a standard 2D angiography [5, 6]. However, whether the software actually influences the number of image acquisitions and overall procedural time has not yet been investigated.

Our current study demonstrated that the feeder-vessel detection software reduced both the total number of image acquisitions and the overall procedural time in TACE of HCC without influencing the therapeutic efficacy. The software can identify a tumor feeder, even from a nonselective C-arm CT arteriography and thus facilitates direct placement of a microcatheter into the feeder vessels without the need for sequential angiography. With this particular capability, the software can potentially reduce radiation exposure and contrast material use, though we did not evaluate these factors in this study. 

It is sometimes difficult to recognize a target location with angiography alone, if the target tumor is small or less enhanced [8–11]. However, these tumor factors do not interfere with the software analysis because C-arm CT has an adequate sensitivity for detecting small and less enhanced HCC lesions [10–13]. Using software for these lesions can avoid sequential catheterizations into all possible arterial branches toward the tumor area and spare a patient from unnecessary additional imaging acquisitions. 

Angiographic assessment of multiple tumor feeders supplying a single lesion usually requires multiple image acquisitions for each feeder. However, the software can determine multiple tumor feeders simultaneously in a single process from the nonselective C-arm CT data. Even in patients with multiple tumors, each tumor feeder can be determined from a single acquisition of a nonselective C-arm CT image during contrast injection from the common or proper hepatic artery. Multiple software processes are necessary to detect each tumor feeder supplying a tumor. A radiology technologist can alternatively participate in the software analysis and thus spare the operating angiographer from procedural interruption. The software also helps to determine the optimal working angle. If it is difficult to ascertain the origin or vasculature of tumor feeders because of overlapping on the frontal image, an optimal 3D roadmap in association with color-coded feeder vessels at any angle can be optimized for better viewing without obtaining additional oblique angiography. With this capability, the software can potentially reduce radiation exposure and contrast material use. 

However, the software has several fundamental limitations. It was designed for selective transcatheter therapy; therefore, its benefit is limited in lobar or whole-liver embolization in patients with numerous or infiltrative tumor burdens. Our current data showed that the total image acquisition and the overall procedural time were indeed slightly reduced in patients with multiple lesions; however, the difference did not reach a statistical significance, even with the use of software. Furthermore, the input imaging data originates from the C-arm CT data obtained from the common or proper hepatic artery; therefore, arteries originating from a more proximal contrast injection site cannot be depicted. Therefore, the software would not recognize tumor feeders arising from the extrahepatic arteries, such as the left gastric artery or the phrenic artery. An angiographer can recognize an extrahepatic supply to a certain hepatic area by detecting the absence of perfusion on a nonselective C-arm CT image. However, manual survey for extrahepatic feeder vessels would be necessary. Furthermore, approximately 10% of the feeder arteries indicated by software analysis are false feeders [3, 5–7]. If an investigated vessel is identified as false, the subsequent placement of a microcatheter into other probable feeder vessels should be performed without the guidance of software analysis. 

This study had a few limitations. First, it was a retrospective comparative analysis. The results of this study may include selection and information biases. Second, despite the decreased number of total image acquisitions with the software, the radiation exposure to the patients or the operators was not evaluated in this study. Third, we were unable to assess the total amount of contrast material used in a single-session TACE because we had not recorded the dose of the contrast material used. Fourth, we did not use 3D images of the C-arm CT for assessing tumor feeders in software-free TACE. The use of such imaging in association with angiography may have improved the detection of tumor feeders in those patients who were analyzed without using software. Fifth, not all tumor feeders were assessed with software analysis, especially those patients who had lobar TACE for multiple tumors. This may have influenced the number of image acquisitions and the procedural time. Sixth, we excluded the study patients who had infiltrative tumors or tumors with an extrahepatic supply. This exclusion made the study patient group somewhat unusual for a daily clinical setting. 

In conclusion, the use of a C-arm angiographic system equipped with automated feeder-vessel detection software in TACE of HCC helped to reduce the number of total image acquisitions and the overall procedural time while maintaining a comparable treatment efficacy, as compared to that of TACE without software assistance.

## Figures and Tables

**Table 1:** Baseline patient and tumor characteristics.

	TACE		*P*
	With software	Without software	
Gender (female/male)	21/29	29/55	0.390
Age (years)*	73 (37–90)	71 (35–89)	0.079
HBs antigen (positive/negative)	5/45	15/69	0.220
HCV antibody (positive/negative)	38/12	60/24	0.567
Child-Pugh class (A/B)	42/8	60/24	0.100
TNM stage (I/II/III)	12/27/11	24/44/16	0.537
Serum AFP level (ng/mL)*	609 (3–12424)	173 (3–3557)	0.191
Previous treatment (primary/recurrence)	13/37	23/61	0.864
Number of tumors (1/2/3/4/5 or greater)	19/10/6/7/8	42/12/10/8/12	0.258
Maximum tumor size (mm)*	21 (9–47)	21 (8–61)	0.623
Number of treatment areas in a single session*	1.7 (1–4)	1.6 (1–4)	0.329
Treatment area (distal/subsegment/segment/lobe)	20/47/11/9	21/72/22/19	0.111

*Data are expressed as the mean (range).

TACE: transarterial chemoembolization.

HB: hepatitis B; HCV: hepatitis C virus; AFP: *α*-fetoprotein.

**Table 2:** Number of image acquisitions required for a single session of chemoembolization.

Imaging	Tumor multiplicity	Number of image acquisition		*P*
		TACE with software (*n* = 50)	TACE without software (*n* = 84)	
Angiography	Single (*n* = 61)	3.7 ± 1.0 (2–6)	6.3 ± 1.7 (3–10)	<0.001
	Multiple (*n* = 73)	5.2 ± 1.9 (2–10)	7.0 ± 2.4 (4–14)	0.001
	Overall	4.6 ± 1.7 (2–10)	6.6 ± 2.1 (3–14)	<0.001

C-arm CT	Single (*n* = 61)	3.4 ± 1.3 (2–7)	3.4 ± 1.5 (2–8)	0.884
	Multiple (*n* = 73)	4.4 ± 1.4 (2–7)	4.0 ± 1.2 (2–7)	0.215
	Overall	4.1 ± 1.4 (2–7)	3.8 ± 1.4 (2–8)	0.228

Total	Single (*n* = 61)	7.1 ± 1.8 (5–12)	9.7 ± 3.0 (5–17)	0.001
	Multiple (*n* = 73)	9.6 ± 2.7 (5–16)	11.0 ± 3.2 (6–20)	0.105
	Overall	8.7 ± 2.7 (5–16)	10.4 ± 3.2 (5–20)	0.004

Data are expressed as mean ± standard deviation (range).

TACE: transarterial chemoembolization.

C-arm CT: C-arm computed tomography.

**Table 3:** Procedural time required for a single chemoembolization.

Tumor multiplicity	Procedural time (min)		*P*
	TACE with software (*n* = 50)	TACE without software (*n* = 84)	
Single (*n* = 61)	88 ± 22 (44–132)	109 ± 29 (58–187)	0.013
Multiple (*n* = 73)	112 ± 35 (50–178)	123 ± 32 (67–228)	0.219

Overall	103 ± 33 (44–178)	116 ± 31 (58–228)	0.021

Data are expressed as mean ± standard deviation (range).

TACE: transarterial chemoembolization.

**Table 4:** Therapeutic efficacy of chemoembolization of hepatocellular carcinoma.

	TACE with software (*n* = 50)	TACE without software (*n* = 84)	*P*
CR	34	53	0.728
PR	5	15	
SD	6	8	
PD	5	8	

CR rate (%)	68	63	0.565
OR rate (%)	78	80	0.680

The response was evaluated according to the modified Response Evaluation Criteria in Solid Tumors [4].

CR: complete response; PR: partial response; SD: stable disease; PD: progressive disease; OR: objective response; TACE: transarterial chemoembolization.
